# Consensus Statements on the Definition of Surgical Success Following Obstetric Urinary Pelvic Floor Fistula Repair: An IUGA-ICS Proposal

**DOI:** 10.1007/s00192-025-06413-6

**Published:** 2026-03-18

**Authors:** L. P. Maljaars, J. Corcos, G. Ghoniem, J. T. W. Goh, T. J. Greenwell, D. Kupualor, R. Pope, S. Rachaneni, M. C. Regmi

**Affiliations:** 1 Department of Obstetrics and Gynecology, Amsterdam UMC location University of Amsterdam, Meibergdreef 9, 1105 AZ Amsterdam, The Netherlands; 2 Amsterdam Reproduction and Development research institute, Amsterdam, The Netherlands; 3 Department of Urology, Jewish General Hospital, McGill University, Montreal, Quebec Canada; 4 Department of Urology, University of California, Irvine, CA USA; 5 Griffith University School of Medicine, Gold Coast, Queensland Australia; 6 Department of Urology, University College London Hospitals, London, UK; 7 Department of Obstetrics and Gynecology, Korle Bu Teaching Hospital, Accra, Ghana; 8 Urology Institute, University Hospitals Cleveland Medical Center, Cleveland, OH USA; 9 Department of Urogynecology, Shrewsbury and Telford Hospitals NHS Trust, Shropshire, UK; 10 Department of Obstetrics and Gynecology, B. P. Koirala Institute of Health Sciences, Dharan, Nepal

**Keywords:** Outcome measurements, Quality of life, Surgical success, Urinary incontinence, Urinary pelvic floor fistula, Vesicovaginal fistula

## Abstract

**Introduction:**

Standardized definitions for surgical success after obstetric urinary pelvic floor fistula (UPF) repair are lacking. This study aimed to establish a consensus among fistula surgeons on defining surgical success for UPF repair caused by obstetric (childbirth-related) injuries.

**Materials and Methods:**

A working group was initiated by the International Urogynecology Association (IUGA) in collaboration with the International Continence Society (ICS). Following a systematic literature review, statements were developed, and a Delphi method was applied to reach a consensus on each statement.

**Results:**

Consensus was reached on 34/44 statements (77.3%). These were grouped into five categories: (1) definition of outcome, (2) treatment and outcome assessment, (3) post-fistula repair urinary incontinence, (4) incurable fistula, and (5) quality of life.

**Discussion:**

The consensus group recommends defining surgical success as anatomical closure of the fistula without residual urinary incontinence, as closure alone is insufficient. Women with residual urinary incontinence should not be classified as cured and require further diagnostic and therapeutic intervention. A postoperative dye test at catheter removal is advised as an objective measure of surgical success. The group also underscores the importance of basic urodynamic evaluation to assess residual incontinence following fistula closure. A diagnosis of “incurable” fistula should only be considered after three failed repairs and requires independent assessments by two expert surgeons. Finally, clinical success should include improvements in patient quality of life, and a specialized, validated quality-of-life questionnaire is essential to evaluate the physical, social, and emotional impact of UPF on patients and to assess treatment effectiveness from the patient’s perspective.

**Conclusion:**

The consensus statements aim to standardize the definition of successful outcomes in obstetric UPF repair, guiding future research and patient counseling. The group encourages further investigation into existing knowledge gaps in obstetric urinary pelvic floor fistula.

**Supplementary Information:**

The online version contains supplementary material available at 10.1007/s00192-025-06413-6.

## Introduction

Obstetric urinary pelvic floor fistula (UPF) (predominantly vesicovaginal fistula (VVF)), particularly complex cases, present a significant surgical challenge in large parts of the world, with repair outcomes far from satisfactory. Surgical repair involves mobilization of the vagina from urethra and/or bladder to allow a tension-free closure. Where possible, a two-layered closure is performed. Fistula repair is often complicated by the presence of extensive tissue loss, scarring, reduced vascularity, and tissue fragility. Limited viable tissue availability may necessitate compromises in vaginal length, diameter, or bladder capacity to permit sufficient tissue mobilization to achieve a tension-free closure. Such adjustments can impact urethral integrity (potentially leading to urinary incontinence), reduce bladder capacity (causing urinary frequency), and shorten vaginal dimensions (which may affect sexual function, conception, and labor) [1, 2].

The aim of UPF surgery is to restore the urinary, sexual, and reproductive functions of affected women [3]. However, there is no standardized definition of surgical success following obstetric UPF repair. Most studies define success as complete anatomical fistula closure and restoration of urinary continence, with reported success rates between 54 and 98% for closure and between 0 and 97% for continence [4–10]. However, definitions and assessment methods vary widely, limiting comparisons across surgical techniques and patient populations. Moreover, current definitions often omit the patient’s perspective, a critical aspect given the multifaceted impact of obstetric fistulas on physical, psychological, social, and economic well-being. A holistic definition of “cure” should incorporate both clinical outcomes and the patient’s perspective. Establishing a consensus on surgical success criteria will enable better treatment comparisons, identification of success predictors, improved patient counseling, and ultimately result in enhanced quality of life for patients.

Defining surgical success in obstetric UPF repair is challenging for several reasons, each of which is crucial to consider when attempting to reach a consensus on the definition of surgical success. First, diagnostic resources are often limited in settings where obstetric fistula repair is performed, resulting in the use of a variety of assessment methods with differing reliability. Second, follow-up of patients is difficult in resource-limited settings due to factors such as poverty, low literacy rates, lack of postal addresses, and unreliable or nonexistent electricity for telephone contact. Consequently, success is often assessed only in the short term. Third, there is no standardized tool to evaluate quality of life post-repair, an essential subjective component of surgical success. Few studies report on this aspect, and those that do often use tools that lack validation for this health problem in the specific context of this patient group.

This work aimed to establish a consensus among fistula surgeons on defining surgical success after UPF repair specifically for cases resulting from birth-related trauma (obstetric UPF). Given the multiple challenges in fistula repair, the criteria for success and outcome assessment should be reliable, feasible, cost-effective, and easily applicable across diverse healthcare settings.

## Methods

A working group was initiated by the International Urogynecology Association (IUGA) and the International Continence Society (ICS) to develop a consensus on defining surgical success for obstetric UPF repair. An open call for applications was made via email and the IUGA and ICS websites, inviting members of both organizations who met the following criteria:

1. Clinical experience in fistula repair surgery,
2. Prior experience in reviewing scientific literature and scientific writing,
3. Commitment to collaboration and task completion.

Applicants submitted their qualifications, and a brief CV. Eight qualified candidates were selected by the Research and Development Committees of the societies, with final approval from each societies’ Board. Appendix A provides a background on each consensus group member and their contributions to the scientific literature.

The development of this clinical consensus used a modified Delphi method, adapted from the clinical consensus statement development manual of the American Academy of Otolaryngology – Head and Neck Surgery Foundation (AAO-HNSF) [11]. A systematic review of the literature was conducted to identify relevant studies on defining surgical success in obstetric UPF. The focus was on obstetric fistulas, given their distinct pathophysiology related to pressure necrosis, as compared to iatrogenic fistulas from surgeries or radiation. Given the high incidence of obstetric fistulas in rural areas with limited surgical access, standardizing surgical success criteria is especially important for the field of obstetric fistula repair.

The search strategy and the flowchart of selected studies can be found in the supplementary materials (Table S1 and Fig. S1). The consensus group reviewed the selected studies and formulated topic questions addressing controversial clinical issues, care variability, evidence gaps, and quality improvement in defining success post-fistula repair. The questions were prioritized, and statements were drafted for the most important topics. On the basis of these drafts, the chair prepared the statements for the Delphi survey. It included two survey rounds, where members anonymously rated each statement on a 9-point Likert scale, from “strongly disagree” (1) to “strongly agree” (9). Members provided comments and suggestions via a text box accompanying each statement. Following each survey, an online meeting was held to discuss results and revise statements to enhance consensus. Modified statements were then re-evaluated in the subsequent survey round. After the second round the last modifications were agreed on in the final online meeting.

Consensus was defined as a mean score of ≥ 7.0 with no more than one outlier, while near consensus was a mean score of ≥ 6.5 with up to two outliers. If the mean score was < 6.5 or there were more than three outliers, consensus was not reached [11]. Unanimity was not required, but statements reflected the general view of the group. Members were also invited to summarize the literature for each statement and explain its rationale.

## Results and Discussion

All members drafted statements and voted on the consensus statements using the Delphi process. The chair did not vote but reviewed and summarized the survey result for the online meeting and modified statements based on group discussion for the subsequent round. A total of 44 statements were included in the survey, with agreement reached on 34 (77.3%) statements. These 34 consensus statements are presented in Table 1, while the 10 statements without consensus are listed in Table 2. The statements were grouped in five categories:

1. Definition of outcome
2. Treatment and outcome assessment
3. Post-fistula repair urinary incontinence
4. Incurable fistula
5. Quality of life

**Table 1:** Statements that reached consensus

Statement	Conclusion	Mean score	Outliers
**Definition of outcome**			
3: The definition of success following surgical repair of vesicovaginal fistula (VVF) is defined as anatomical closure without residual incontinence.	Consensus	7.5	0
42: Closing the hole (fistula) is not good enough as an outcome, since the whole individual needs to be assessed.	Consensus	7.0	0
16: Clinical success after surgical VVF repair is defined as the resolution of symptoms (e.g., urinary incontinence, pain and sexual dysfunction) and absence of any signs of VVF on physical examination or imaging.	Consensus	8.0	1
20: Future pregnancy and reproductive function are not considered a primary outcome of fistula repair since more factors determine reproductive function than the fistula.	Consensus	8.4	0
**Treatment and outcome assessment**			
22: Setting outcome goals is the most important step in managing patient's expectations.	Consensus	8.5	0
35: The best VVF closure techniques restore the normal genitourinary anatomy and considers bladder function.	Consensus	9.0	0
36: Flap splitting techniques can be used in VVF cases with less risk of reducing vaginal capacity.	Consensus	7.6	0
2: Fistula closure should be assessed with a postoperative dye test at time of catheter removal (10–14 days after surgery). The dye test includes infusion of dye through the catheter into the bladder. Any leakage can be assessed under direction vision during vaginal examination or by vaginal insertion of a tampon/pad after filling the bladder. A prolonged period of ambulation is not required as long as the bladder is sufficiently full with 200–300 mL of fluid.	Consensus	7.6	1
21: Appropriately trained medical staff (trained nurses and non-consultant grade medical staff) can perform a postoperative dye test when the surgeon is unable to follow up on the results.	Consensus	7.4	1
37: Following obstetric VVF repair, continuous bladder drainage via catheter lasts for a minimum of 10–14 days.	Consensus	7.9	1
14: Large fistula size (>3cm), number of fistulas, scar tissue, urethral involvement, distance to the ureteric orifices and small bladder capacity (<100ml) affects anatomical closure with urinary continence negatively.	Consensus	8.9	0
15: A fistula that breaks down within 3–6 months is considered as a failed repair.	Near consensus	6.6	2
**Post-fistula repair urinary incontinence**			
11: Complete history, examination, post-void residual, cough-test, urinary diary and (simple) urodynamics are part of evaluation of post-fistula repair urinary incontinence.	Consensus	8.1	1
40: Simple urodynamic evaluation is a necessary tool in assessing residual incontinence after successful closure of the fistula.	Consensus	7.0	1
12: Pelvic floor muscle training is beneficial for women with stress urinary incontinence.	Consensus	8.0	0
23: Pelvic floor muscle training (PFMT) is the preferred conservative treatment of post-repair residual stress urinary incontinence.	Consensus	7.5	1
13: Bladder and pelvic floor muscle training (PFMT) and anti-spasmotics can be beneficial for women with urgency incontinence.	Consensus	9.0	0
**Incurable fistulas**			
38: Women are deemed incurable if they have persisting or recurrent fistula for which anatomical repair is not possible.	Consensus	8.4	0
7: Incurable fistulas are not amenable to satisfactory closure with a reasonable number of surgeries, judged by at least two expert surgeons.	Consensus	8.0	0
8: Incurable is defined as women with primary, persisting and recurrent fistula for which anatomical repair is not possible.	Consensus	8.3	0
9: Diversion can be beneficial for incurable patients, when considering the acceptance and availability of devices.	Consensus	8.3	1
**Quality of life**			
1: The definition of clinical success following surgical repair of VVF is measured by patients’ improvement quality of life.	Consensus	8.1	0
4: Improvement in quality of life is the best patient-reported outcome measurement (PROM) for surgical repair of VVF.	Consensus	7.8	1
18: Validated quality of life questionnaires are useful tools for assessing the impact of obstetric VVF on patients’ physical, social, and emotional well-being, as well for evaluating the effectiveness of treatments and interventions.	Consensus	8.9	0
6: Subjective measures of patient's outcome include sexual satisfaction, psychological well-being, pain scale and social reintegration.	Consensus	8.5	0
5: PROM for VVF surgery include complete urinary continence, complete volitional voiding and normal sexual function.	Consensus	7.1	1
28: The Patient Global Impression of Improvement (PGI-I) is suitable to assess the patient's overall satisfaction after fistula repair.	Consensus	8.4	0
30: The Incontinence Quality of Life (I-QOL) questionnaire is suitable to assess urinary incontinence and the effect of symptoms, daily activities, emotions and social functioning after fistula repair.	Consensus	7.6	1
31: The Urinary Distress Inventory (UDI) is suitable to assess urinary symptoms, such as frequency, urgency, and leakage after fistula repair.	Consensus	8.3	0
44: The International Consultation on Incontinence Questionnaire-Urinary Incontinence Short Form (ICIQ-UI SF) is suitable to assess the frequency, severity and impact on quality of life (QoL) of urinary incontinence after fistula repair.	Consensus	7.6	0
41: Quality of life of patients should be assessed 3–12 months after fistula repair.	Consensus	7.6	1
19: Sexual function after fistula repair should be assessed by a validated questionnaire at 6–12 months after the repair.	Consensus	8.6	0
17: The domains of quality of life (QoL) for women living in a small community in a low- and middle-income country (LMIC) may vary depending on cultural and contextual but include health, social support, education, economic status, environment, cultural and spiritual beliefs and gender roles and equality.	Consensus	7.4	1
43: A specialized and validated short questionnaire is needed for fistula to assess quality of life.	Consensus	8.8	0

**Table 2:** Statements that reached no consensus

Statement	Conclusion	Mean score	Outliers
10: Urodynamic evaluation can distinguish between women where anatomical closure is not possible and women who have a closed fistula but are severely incontinent.	No consensus	4.5	5
24: Rectus fascial sling is the preferred surgical treatment of post-repair residual stress incontinence.	No consensus	6.4	5
25: Women need to be able to perform intermittent self-catheterization (ISC) before performing rectus fascial sling (RFS) surgery. Reusable metal catheters and advice on how to store and clean these catheters is necessary.	No consensus	4.9	5
26: Intravesical botulinum toxin, if available, is a last resort for women with post-repair urgency urinary incontinence despite PFMT and/or anticholinergics.	No consensus	5.0	6
27: Women need to be able to perform intermittent self-catheterization (ICS) before receiving intravesical botulinum toxin. Reusable catheters and advice on how to store and clean these catheters is necessary.	No consensus	7.1	3
29: The King's Health Questionnaire (KHQ) is a suitable tool assess symptoms of urinary continence, daily activities, emotions, and social functioning of patients after fistula repair.	No consensus	5.9	5
32: The Pelvic Organ Prolapse/Urinary Incontinence Sexual Questionnaire (PISQ-12) is suitable to assess the impact of urinary symptoms on sexual function after fistula repair.	No consensus	5.6	5
33: The Short Form (SF-36) is a suitable tool to a assess physical and mental health, including questions about physical functioning, social functioning, role limitations due to physical or emotional problems, and pain after fistula repair.	No consensus	5.0	6
34: The EuroQoL 5-Dimension (EQ-5D) is a suitable standardized instrument for measuring health outcomes including questions about mobility, self-care, usual activities, pain/discomfort, and anxiety/depression after fistula repair.	No consensus	6.4	4
39: Women are deemed incurable who have a closed fistula but are severely incontinent.	No consensus	3.9	3

We begin by defining surgical success and outlining the criteria for a successful repair. Following this, we discuss treatment methods and how outcomes are measured through clinical evaluations, imaging, and other diagnostic tools. Post-repair urinary incontinence, a significant concern, is addressed next, with a focus on targeted assessment and management. The section on incurable fistulas follows, examining complex cases where repair may not be possible. The concluding section focuses on assessing the patient’s quality of life, which is the ultimate measure of repair success. These tools provide a holistic view of the outcomes beyond clinical indicators and help assess the long-term success of treatments.

### Definition of outcome

The consensus group recommends that the definition of success following surgical repair of obstetric UPF should include not only successful anatomical closure but also the absence of residual urinary incontinence [statement 3]. Urinary continence is achieved when the woman who previously had UPF related urinary incontinence has no urine leakage after repair. A recent survey of expert fistula surgeons showed that ongoing urinary incontinence was viewed as the most significant factor in determining cure [12]. This aligns with the consensus group’s assertion that closing the fistula alone is insufficient, as a holistic assessment of the patient’s physical and psychological health is essential [statement 42]. This is also in accordance with the aforementioned survey among expert fistula surgeons [12]. The writing group acknowledges that residual symptoms do not negate the surgical/anatomical achievement, but propagates a broader, more holistic approach.

#### Anatomical Closure and Urinary Continence Rates

Studies commonly report surgical success as successful anatomical closure, with success rates ranging from 54% to 98%. However, rates of urinary continence following these “successful” closure are typically lower, ranging from 0% to 96.8% [4–10]. Approximately one in four women (range 3.2–100%, depending on fistula size, site, and special factors such as scarring) report some degree of persistent urinary incontinence at discharge from hospital after successful anatomical closure of their fistula [6, 7, 9, 13]. The disparity between anatomical closure and continence is known as the “continence gap,” which affects an estimated 15% to 20% of women after obstetric fistula repair [10]. It should be noted that de novo bladder storage issues (e.g., overactive bladder syndrome) as well as stress urinary incontinence contribute to the continence gap.

#### Clinical Success Beyond Anatomical Closure

The group highlights that obstetric fistulas result from “field injuries” affecting a broad area of the pelvis. This can include urethral loss, stress urinary incontinence, vaginal stenosis, sexual dysfunction, secondary amenorrhea, secondary infertility, cervical incompetence (as a consequence of cervical scarring or destruction), rectovaginal fistula, anal sphincter incompetence, fecal incontinence, rectal atresia, hydroureteronephrosis, renal failure, osteitis pubis, and footdrop. In addition to these physical dysfunctions, there are well-described social and emotional/psychological consequences of UPF which should also be considered. Persistent urinary incontinence is also associated with significant psychological and social impacts, emphasizing the need for a comprehensive approach to defining success [6, 14–18]. It therefore follows that complete anatomical closure alone is not an adequate parameter regarding the outcome of obstetric fistula repair [6, 19].

#### Assessing Clinical Success Through Symptom Resolution

To achieve a more comprehensive outcome measure, the consensus group recommends defining clinical success not solely by anatomical closure but also by the resolution of key symptoms (e.g., urinary incontinence, pain, sexual dysfunction) and the absence of urinary pelvic floor fistula signs on examination or imaging [statement 16]. When assessing symptom resolution, it is important to acknowledge that the multifaceted trauma from prolonged labor (e.g., urethral involvement, circumferential defects, neurogenic trauma to pelvic floor) may have other manifestations such as stress urinary incontinence which will affect the patient’s perception of cure. Patients must be informed that achieving anatomical closure may not fully resolve urinary incontinence, and additional treatments may be needed. Cases with residual urinary incontinence (i.e., urine leakage, either patient reported or on clinical examination) with or without anatomical closure should not be deemed cured since symptoms have not been resolved and further diagnostics and treatment should be considered [6, 7, 9].

#### Reproductive Health After Fistula Repair

Though reproductive health is a concern for many women post-fistula repair, the group advises that reproductive outcomes should not be considered primary indicators of success due to the multifactorial nature of fertility issues [statement 20] [16]. While amenorrhea affects 5% to 44% of women pre-repair, menses resumes in most post-repair cases. Conversely, 7% to 42% will experience unexplained amenorrhea afterward [20, 21]. Only 17.4% (range 2.5–40%) of women become pregnant after repair, with most pregnancies resulting in term deliveries, although some studies report increased rates of miscarriage and stillbirth [21–23]. Secondary infertility occurs in 5% to 60% [20, 21, 23]. Possible causes of secondary infertility include uterine/cervical adhesions, post-partum pituitary necrosis, functional hypothalamic amenorrhea, not having a sexual partner, or unexplained.

### Treatment and Outcome Assessment

#### Treatment Approach

The consensus group emphasizes the importance of presurgical counseling to set realistic expectations for fistula repair. Patients should be fully informed about their condition, treatment options, and expected outcomes, enabling the surgeon and patient to agree on achievable goals. Defining the outcome goal is an important step in managing patients’ expectations [statement 22].

Since the genitourinary system comprises the kidneys, ureters, bladder, urethra, and genital organs, an optimal fistula repair surgery takes the anatomy and function of these organs into consideration, especially bladder function [statement 35]. Proper evaluation of the genitourinary anatomy is essential before surgery, as post closure variations can affect patient satisfaction despite a successful anatomical closure. Any adverse findings should be discussed with the patient to optimize outcomes. Fistula closure can be achieved through the vaginal or abdominal route. The most common vaginal approaches are the flap splitting technique and the Latzko repair [24–26]. The Latzko repair has been reported to produce postoperative vaginal shortening which can be associated with difficulties in vaginal intercourse [27]. Thus, the Latzko repair may not be suitable for cases with pre-existing short vaginal length. The flap splitting technique has a smaller risk of reducing vaginal capacity and is a better technique in patients with short vaginal length [statement 36]. The two closure techniques (flap splitting and Latzko) are equally effective in terms of successful UPF closure with similar published surgical outcomes (although no comparative studies have been published).

#### Postoperative Dye Test

Routine postoperative care should include a dye test at catheter removal to confirm anatomical closure [statement 2]. This allows the surgeon to differentiate between a failed repair and other causes of urinary incontinence [28]. A dye test involves filling the bladder with a safe medical dye (e.g., dilute gentian violet, methylene blue or contrast), allowing direct visualization of any leakage or, if unclear, using a tampon after dye instillation for high (proximal) fistulas [24, 29, 30]. This procedure may be performed by suitably trained nurses or other clinical staff if the surgeon is unavailable [statement 21] [24]. If available, a retrograde cystogram can be performed as an alternative, more objective measure since a dye test can be subject to observer variation. However, in many parts of the world, this is not available, so a dye test suffices.

#### Postoperative Catheter Management

The consensus group agrees unanimously that continuous bladder drainage via transurethral catheter is mandatory after UPF repair to aid tissue healing and prevent repair breakdown. The optimal catheterization duration remains to be determined, with the WHO recommending 7–10 days [31]. Studies show that 7 days may suffice for simple cases, although complex fistulas may require more extended drainage [29, 32]. On the basis of earlier work, the consensus group recommends a minimum catheter duration of 10 to 14 days [statement 37] [33].

#### Early Surgical Failure

Factors that negatively impact surgical success include large fistula size (>3 cm), multiple fistulas, scarring, urethral involvement, ureteric involvement, close proximity to the ureteric orifice and/or a small bladder capacity (<100 ml) [statement 14] [9, 34–37]. Patients with larger fistulas have a higher likelihood of early repair failure due to tissue mobilization challenges and having insufficient tissue to achieve tension-free repair [38]. Scar tissue and small bladder remnants reduce successful outcomes and can limit eventual bladder capacity and function [5, 7, 35, 37]. Ureteral involvement may necessitate ureteral reimplantation, which can lead to long-term renal impairment. Preventive measures to avoid early breakdown are bladder decompression by urethral catheterization, sufficient fluid intake to prevent stone formation, antibiotics to prevent infection, and abstinence from sexual intercourse.

#### Delayed Surgical Failure

The exact time frame for fistula breakdown after an apparent initially successful closure remains unknown. Recurrence of the fistula defect and urinary incontinence may occur after a period of transient complete fistula healing due to delayed complications with wound healing causing the fistula repair to breakdown and reform [30]. Successfully repaired fistulas have been reported to breakdown from 10 days to 6 months post-surgery in the absence of new risk factors such as a new pregnancy [32, 39]. Delayed surgical failure may occur in women who conduct vigorous physical activity and/or have returned to sexual intercourse [40]. On the basis of expert opinion, a fistula that breaks down/fails within 3–6 months is considered a failed repair [statement 15]. This is not a new fistula.

### Post-Fistula Repair Urinary Incontinence

#### Evaluation of Post-Fistula Repair Urinary Incontinence

The consensus group recommends a comprehensive evaluation of urinary incontinence after fistula repair, which should include a detailed medical history, a physical examination, measurement of post-void residual urine, a cough-test, and a bladder diary [statement 11]. Urodynamic testing should be reserved for cases where invasive treatment is considered. The assessment approach is similar to that for women without a fistula history. Urinary leakage may result from a weakened external sphincter (stress urinary incontinence), overactive bladder (urgency incontinence), or incomplete bladder emptying (overflow incontinence). Overactive bladder symptoms are an often-overlooked issue that contributes to the previously mentioned “continence gap” after fistula repair. Overflow incontinence can sometimes arise following urethral reconstruction or fascial sling procedures conducted during fistula repair. European, US, and other guidelines emphasize the importance of a thorough incontinence history and comprehensive physical examination, which includes assessing for prolapse, scars, deformities, and any leakage during coughing or straining, along with measuring post-void residual urine [41–43]. Assessing post-fistula incontinence requires an in-depth evaluation of both bladder and urinary sphincter function. Cystoscopy and basic urodynamic testing are essential. Basic urodynamics can be performed using a simple urinary catheter connected to a water column and sterile solution [30, 44, 45]. The consensus group recognizes that basic urodynamic testing is crucial for assessing residual incontinence after successful closure of fistula [statement 40] if further treatment is required. Although advanced multichannel urodynamic equipment may be lacking in areas where fistulas are prevalent, simpler tools can still provide reliable information. For instance, a voiding diary, maintained with a basic measuring device like a modified 2L water bottle, can offer valuable insights into voiding frequency and bladder capacity. Using basic tools such as a Foley catheter, Toomey syringe, and saline solution, bladder pressure during filling, the presence or absence of detrusor overactivity, bladder compliance, and cystometric capacity can be evaluated [46]. Leak point pressure can be estimated after catheter removal through progressive Valsalva or cough tests. In addition international guidelines also recommend urodynamic testing before any surgical intervention, particularly in complex cases where symptoms are hard to describe [41].

#### Treatment of Post-Fistula Repair Stress Urinary Incontinence

The consensus group agrees that pelvic floor muscle training (PFMT) is effective for women with post-repair stress urinary incontinence [statement 12] and supports its use as the preferred conservative treatment [statement 23]. Research indicates that PFMT, either alone or in combination with biofeedback or electrostimulation, can reduce incontinence and strengthen pelvic floor muscles and is the most effective nonsurgical option for incontinence [47, 48]. Although PFMT can be highly effective, compliance is critical to maintain benefits [49]. In low-resource settings, alternative methods like “Paula exercises” have been found helpful for managing post-fistula incontinence [50]. A study in Benin found that PFMT improved fistula closure rates and reduced stress incontinence after 1 year [51]. This study also showed that preoperative physiotherapy may contribute positively to surgical recovery and may result in lower rates of postoperative stress urinary incontinence.

#### Treatment of Post-Fistula Repair Urgency Incontinence

For urgency incontinence, combining bladder and pelvic floor muscle training with anticholinergics or β3-agonists can be effective [statement 13]. Although studies differ in terms of interventions, populations and outcomes, PFMT consistently shows greater efficacy than no treatment. Women undergoing PFMT report fewer leaks, perform better on pad tests, void less frequently, and experience improved sexual function [52]. However, the quality of evidence is limited by small sample sizes, short study durations, and the absence of large, well-controlled trials. The efficacy of medications like anticholinergics and β3 agonists is well-established and supported by robust evidence [43, 53, 54].

### Incurable Fistula

The diagnosis of an incurable fistula should be considered after three failed fistula repairs where successful anatomical repair has not been possible despite adequate surgical technique and postoperative care (including the use of prophylactic and therapeutic antibiotics, adequate length of catheterization, adequate operative documentation on technical difficulties encountered during surgery, adequate surgeon training and case load, and full patient compliance with postoperative care advice) [statement 38]. This challenges the surgeon to carefully consider when not to operate or to refer and to protect patients from unnecessary, fruitless and harmful repair attempts. The patient and their fistula will need to be independently assessed by two expert fistula surgeons prior to being declared incurable [statement 7]. A clear definition of an expert surgeon is lacking, and different parameters are used to determine competency level (e.g., number of repairs, duration of training, ability to perform specific types of surgical procedures). The latest version of the FIGO training manual requires 200–300 repairs to be level 2 competent where level 3 competency involves not a specific number of repairs but the ability to perform procedures for urinary diversion, colonic neovagina, circumferential/stenosed rectovaginal fistula and the principles of managing a fistula treatment facility [55]. The older manual described 15 performance-based competences with a minimal training duration of 2 years [56]. In his training manual, Dr. Waaldijk deems surgeons who have performed 250–300 repairs suitable for advanced training to become a future trainer [57]. A recent study that interviewed expert fistula surgeons defined an expert surgeon as a person who has completed >200 fistula surgeries, based on current obstetric fistula training programs [12]. None of these definitions are evidence based—all are opinion based. There is no data on learning curves for obstetric fistula surgery, and this needs to be urgently determined.

A fistula is declared incurable when the conditions for a reasonable chance of success are no longer met [statement 8] [30, 58]. These conditions are related to the size of the fistula, the quality of the bladder and urethral tissue, the residual bladder capacity after closure, the urethral sphincter competence and any other medical conditions that could interfere with a good surgical result. In the absence of an established definition for incurable fistulas–a very complex condition—the consensus group has tried to define the minimal requirements for diagnosing an incurable fistula. We acknowledge that “incurable” is not absolute but relative to the context.

In cases of incurable fistulas, urinary diversion procedures such as ileal conduits, Mainz II pouches, or ureterosigmoidostomies have shown benefits in low-resource settings [statement 9] [59, 60]. However, the availability of equipment, patient acceptance, and long-term follow-up for potential complications (e.g., metabolic, or oncological; in case of conduits or heterotopic pouches) should be thoroughly discussed [61, 62]. Ethical considerations, including the higher risks associated with these invasive procedures, must also be evaluated to ensure patients receive optimal care and outcomes [58, 63, 64]. For example, ureterosigmoidostomy is no longer widely performed in Europe or North America due to high complication rates (e.g., colon cancer). Current long-term data on these procedures in low-resource settings is limited, warranting cautious discussion with patients before attempting urinary diversion.

### Quality of Life

#### Patient-Reported Quality of Life as an Outcome Measure

Obstetric fistula adversely affects mental health, often resulting in depression, anxiety, and even suicide [3, 65–69]. Although quality of life often improves after fistula repair, this is not always true for cases with failed fistula closure, vaginal stenosis, or persistent urinary incontinence [6, 7, 9, 15, 17, 21, 40, 69–77]. The consensus group underscores the importance of measuring clinical success by assessing improvement in patients’ quality of life, as this provides a holistic view of recovery [statement 1]. While surgeon perceptions emphasize anatomical success, incorporating patient perspectives in defining treatment goals and evaluating outcomes is vital [12]. Furthermore, the group emphasizes that quality of life improvement is a key outcome post-repair [statement 4] and endorses validated quality-of-life questionnaires to assess the physical, social, and emotional impacts of obstetric fistula and the effectiveness of treatments [statement 18].

#### Key Aspects of Quality of Life After Fistula Repair

Subjective measures of patient’s outcome, including sexual satisfaction, psychological well-being, pain scores and social reintegration, are crucial indicators of overall success [statement 6].

- **Sexual satisfaction**: Only 35 to 62% of women resume sexual activity after successful fistula repair without residual incontinence, with satisfaction rates of 51% among those sexually active [40, 73, 78]. Reasons for sexual dysfunction include dyspareunia in 9 to 23.5%, lack of sexual desire in 15%, incontinence in 15%, fear of becoming pregnant, fear of sexually transmitted disease and intimate partner violence [2, 21, 40, 68, 73, 78].
- **Psychological well-being**: Persistent urinary incontinence, lack of social (including partner) support, sexual dysfunction, concerns over fertility, and economic exploitation are significant psychological stressors for women post-fistula repair [15, 17, 18, 66, 68, 78–80].
- **Pain scores**: Although data on pain is limited, studies show improved quality of life following fistula repair due to decreased urinary incontinence and relief from perineal pain [80].
- **Social reintegration**: Social acceptance varies by region, with partner abandonment rates between 42% and 66.5% [16, 68, 81]. Reintegration challenges, particularly for women with residual incontinence and divorced women, underscore the need for culturally sensitive support mechanisms.

#### Recommendation for Patient-Reported Outcome Measures (PROMs)

The consensus group recommends using PROMs to assess complete urinary continence, voluntary voiding, and normal sexual function post-fistula surgery [statement 5]. The group recognizes the limited research on PROMs for UPF patients. The ideal PROM tool for obstetric fistula repair is yet to be defined and further studies are urgently needed.

To date, the questionnaires used to assess quality of life vary considerably, making comparison between studies and participants impossible. The most common questionnaire is the abbreviated World Health Organization Quality of Life Brief Version (WHOQOL-BREF) [70, 72, 77, 80, 82]. The WHOQOL-BREF is appealing to use in the obstetric UPF population as it was developed in 15 countries in different regions of the world. It has four domains: physical health, psychological, social relationships, and environmental. Other studies have used the King’s Health Questionnaire [17, 76]. This questionnaire assesses the impact of urinary incontinence on the patient’s quality of life. Unfortunately, it is long and may not be suitable for many low-income communities since this and most other available questionnaires are only validated in high resource countries. In addition, reporting is influenced by education level and expertise, and as such most questionnaires are not suitable for illiterate patients with no formal education. Therefore, the consensus group did not reach consensus on the use of the King’s Health Questionnaire for obstetric fistula patients (Table 2). This was also the case for the Pelvic Organ Prolapse/Urinary Incontinence Sexual Questionnaire (PISQ), the Short Form Health Survey (SF-36), and the EuroQol 5D (EQ-5D).

#### Suitable Quality of Life Questionnaires

Questionnaires deemed suitable by the consensus group for assessing PROM after obstetric fistula repair include:

- The Patient Global Impression of Improvement (PGI-I) – this is a PROM that assesses the patient’s overall satisfaction and is simple, easy, and quick [statement 28]. The Likert scale can be used in any language, so comparisons will be valid from different countries.
- The Incontinence Quality of Life (I-QOL) questionnaire is a PROM that assesses the impact of urinary incontinence on a patient’s quality of life. Although it doesn’t account for cultural aspects, it is suitable to assess urinary incontinence and the impact of symptoms on daily activities, emotions, and social functioning after fistula repair [statement 30].
- The Urinary Distress Inventory (UDI) is a PROM that assesses the severity of urinary symptoms, such as frequency, urgency, and leakage. Its simplicity makes it suitable to use in different countries [statement 31].
- The International Consultation on Incontinence Questionnaire-Urinary Incontinence Short Form (ICIQ-UI SF) is suitable to assess the frequency, severity, and impact on quality of life (QoL) of urinary incontinence after fistula repair [statement 44].

The consensus group recommends QoL assessment at least once between 3 and 12 months post-repair to capture recovery and adaptation [statement 41]. Long-term evaluations over a year provide insights into sustained improvements. Sexual function assessment is also recommended within 6 to 12 months [statement 19].

#### Cultural Sensitivity in Assessing Quality of Life

Cultural sensitivity and recognition of contextual factors are important when assessing quality of life in obstetric fistula patients. These factors are especially critical in countries in Sub-Saharan Africa where cultural exclusion may persist despite successful fistula management. The domains of quality of life for a fistula patient in low- and middle-income countries (LMIC) may vary depending on cultural and contextual factors but some common domains important to consider include health, social support, education, economic status, environment, cultural and spiritual beliefs, and gender roles and equality [statement 17]. These domains may overlap and intersect, and the relative importance of each domain may vary depending on the individual and their context. By considering these domains, healthcare providers and policymakers can gain a better understanding of the factors that contribute to the quality of life for women in small low-income communities and can work to develop interventions that address these factors to improve overall health and well-being.

#### A Specialized Short Quality of Life Questionnaire

Lastly, the consensus group wishes to stress the need for a specialized and validated short questionnaire to assess quality of life for patients with obstetric fistula [statement 43]. A specialized questionnaire is necessary after obstetric UPF repair for several reasons: (1) subjective assessment, to capture the patient’s perspective; (2) tailored patient-centered evaluation, to address specific aspects related to obstetric urinary pelvic floor fistulas; (3) quantitative data, for more objective evaluation of the impact of surgical repair on quality of life; and (4) monitoring progress over time. Overall, a specialized questionnaire enhances the thoroughness and precision of post-fistula repair assessments, leading to a more comprehensive understanding of patient outcomes and facilitating improved clinical care and research in this specialized area.

### Strengths, Limitations, and Future Recommendations

This proposal provides a valuable expert consensus on defining successful surgical repair of UPFs, presenting a practical approach for outcome assessment tailored to low-resource settings. Key strengths include the extensive expertise of the writing group in obstetric fistula management and the geographical diversity represented within the group, which enhances the applicability of the recommendations. The paper proposes a structured framework for evaluation and treatment, supporting standardized care. Its focus on patient-reported outcomes and quality of life emphasizes holistic recovery, while the endorsement of basic, adaptable urodynamic testing expands feasibility in settings with limited access to advanced diagnostic tools.

Nevertheless, the paper has certain limitations. Obstetric fistula research remains limited, and much of the existing evidence is of low quality. This justifies reliance on expert opinion for areas lacking substantial data. Additionally, the consensus process necessarily narrowed its focus to obstetric UPF, excluding other fistula types (e.g., rectovaginal fistulas), and non-obstetric causes (e.g., iatrogenic, or postradiation fistulas). Lastly, while recommendations seek to accommodate diverse contexts, their feasibility may vary, particularly in extremely low-resource settings where basic assessments and quality-of-life measures might be challenging to implement.

Future research directions include the development and validation of a brief quality-of-life questionnaire specific to patients with obstetric UPF, which could further enhance patient-centered care and standardize outcomes assessment in this field.

## Conclusions

Consensus statements were drafted to reach consensus among fistula surgeons on the definition of surgical success after obstetric UPF repair. The consensus group recommends that the definition of success should minimally include anatomical closure without residual urinary incontinence and that anatomical closure of the fistula is not a sufficient outcome. Cases with residual incontinence should not be deemed cured and further diagnostics and treatment should be considered. Furthermore, it recommends measuring clinical success by patients’ improved quality of life and stresses the need for a specialized and validated short questionnaire to assess quality of life for patients with obstetric fistula. Lastly, the consensus group aims to inspire further research into the numerous knowledge gaps surrounding obstetric UPF and genitourinary fistulas in general.

## Supplementary Information

Below is the link to the electronic supplementary material.

Supplementary file1 (DOCX 37.8 KB)

Supplementary file2 (DOCX 49.5 KB)

Supplementary file3 (DOCX 16.8 KB)
