# Supplementary material for: Consensus Statements on the Definition of Surgical Success Following Obstetric Urinary Pelvic Floor Fistula Repair: An IUGA-ICS Proposal
Source: Int Urogynecol J. 2026 Mar 18;37(5):1193–205. doi: 10.1007/s00192-025-06413-6 (PMC13226361; doi:10.1007/s00192-025-06413-6)
Supplement: Supplementary file 1 — Supplementary file1 (DOCX 37.8 KB) [file 192_2025_6413_MOESM1_ESM.docx]

**Search strategy – systematic literature review**

| Source: | Ovid MEDLINE(R) ALL <1946 to January 18, 2022> | Date of search 19-01-2022 |
| --- | --- | --- |
|  |  |  |
| # | Searches | Results |
| 1 | exp Vaginal Fistula/ | 4983 |
| 2 | ((obstetric* or genito?rinar* or genital or vesico* or vaginal) adj3 fistul*).ab,kf,ti. | 4762 |
| 3 | (((midvaginal or juxtacervical) adj3 obstetric vesicovaginal fistul*) or VVF).ab,kf,ti. | 366 |
| 4 | 1 or 2 or 3 [Genitourinary Fistula] | 7112 |
| 5 | exp Surgical Procedures, Operative/ | 3371405 |
| 6 | operative surgical procedure.ab,kf,ti. | 42 |
| 7 | (surg* or repair* or reconstruct*).ab,kf,ti. | 2654009 |
| 8 | 5 or 6 or 7 [Repair] | 4815494 |
| 9 | 4 and 8 [Genitourinary Fistula and Repair] | 4943 |
| 10 | (exp animal/ not human/) or disease models, animal/ or exp animals, laboratory/ or exp rodentia/ or cattle/ or exp animals, genetically modified/ | 5896187 |
| 11 | ((animal adj3 (model* or experiment* or genetically modified)) or canine or dog or dogs or beagle* or feline or cat or cats or rodent* or rabbit* or mice or mouse or murine* or rat or rats).ab,kf,ti. | 3528287 |
| 12 | 10 or 11 [Animal filter] | 6420184 |
| 13 | 9 not 12 [Animal studies excluded] | 4868 |
| 14 | case reports/ | 2240395 |
| 15 | (case serie* or case report* or case stud*).ab,pt,ti. | 2485245 |
| 16 | 14 or 15 [Case reports] | 2485245 |
| 17 | 13 not 16 [Case reports excluded] | 3443 |

| Source: | Ovid MEDLINE(R) ALL <1946 to March 19, 2024> | Date of update  20-03-2024 |
| --- | --- | --- |
|  |  |  |
| # | Searches | Results |
| 1 | exp Vaginal Fistula/ | 5157 |
| 2 | ((obstetric* or genito?rinar* or genital or vesico* or vaginal) adj3 fistul*).ab,kf,ti. | 5141 |
| 3 | (((midvaginal or juxtacervical) adj3 obstetric vesicovaginal fistul*) or VVF).ab,kf,ti. | 432 |
| 4 | 1 or 2 or 3 [Genitourinary Fistula] | 7576 |
| 5 | exp Surgical Procedures, Operative/ | 3603702 |
| 6 | operative surgical procedure.ab,kf,ti. | 55 |
| 7 | (surg* or repair* or reconstruct*).ab,kf,ti. | 3004279 |
| 8 | 5 or 6 or 7 [Repair] | 5286873 |
| 9 | 4 and 8 [Genitourinary Fistula and Repair] | 5263 |
| 10 | (exp animal/ not human/) or disease models, animal/ or exp animals, laboratory/ or exp rodentia/ or cattle/ or exp animals, genetically modified/ | 6227866 |
| 11 | ((animal adj3 (model* or experiment* or genetically modified)) or canine or dog or dogs or beagle* or feline or cat or cats or rodent* or rabbit* or mice or mouse or murine* or rat or rats).ab,kf,ti. | 3790708 |
| 12 | 10 or 11 [Animal filter] | 6815904 |
| 13 | 9 not 12 [Animal studies excluded] | 5184 |
| 14 | case reports/ | 2391470 |
| 15 | (case serie* or case report* or case stud*).ab,pt,ti. | 2681064 |
| 16 | 14 or 15 [Case reports] | 2681064 |
| 17 | 13 not 16 [Case reports excluded] | 3650 |
| 18 | ("23201329" or "22914460" or "27821123" or "33164206" or "33811637" or "31778211" or "29441572" or "27741181" or "26233699" or "20700729").an. | 10 |
| 19 | limit 17 to yr="2022 -Current" | 223 |

| Source: | Embase Classic+Embase <1947 to 2022 January 14> | Date of search: 19-01-2022 |
| --- | --- | --- |
|  |  |  |
| # | Searches | Results |
| 1 | exp cystovaginal fistula/ | 3863 |
| 2 | ((obstetric* or genito?rinar* or genital or vesico* or vaginal or cytovaginal) adj3 fistul*).ab,kw,ti. | 6891 |
| 3 | (((midvaginal or juxtacervical) adj3 obstetric vesicovaginal fistul*) or VVF).ab,kw,ti. | 776 |
| 4 | 1 or 2 or 3 [Genitourinary Fistula] | 8015 |
| 5 | exp surgery/ | 5739398 |
| 6 | (surg* or repair* or reconstruct*).ab,kw,ti. | 3602699 |
| 7 | 5 or 6 [Repair] | 6917281 |
| 8 | 4 and 7 [Genitourinary Fistula and Repair] | 6157 |
| 9 | limit 8 to conference abstracts | 1534 |
| 10 | 8 not 9 [Conference abstracts excluded] | 4623 |
| 11 | (exp animal/ not human/) or animal disease/ or exp experimental animal/ or exp animal experiment/ or exp animal model/ or exp domestic cattle/ or exp rodent/ | 7114556 |
| 12 | ((animal adj3 (model* or experiment*)) or canine or dog or dogs or beagle* or feline or cat or cats or rodent* or rabbit* or mice or mouse or murine* or rat or rats).ab,kw,ti. | 4566751 |
| 13 | 11 or 12 [Animal filter] | 7707225 |
| 14 | 10 not 13 [Animals excluded] | 4555 |
| 15 | exp case study/ | 92538 |
| 16 | (case adj3 (stud* or series)).ab,kw,ti. | 474425 |
| 17 | 15 or 16 [Case reports] | 495804 |
| 18 | 14 not 17 [Case reports excluded] | 4462 |

| Source: | Embase Classic+Embase <1947 to 2024 March 19> | Date of update: 20-03-2024 |
| --- | --- | --- |
|  |  |  |
| # | Searches | Results |
| 1 | exp cystovaginal fistula/ | 4203 |
| 2 | ((obstetric* or genito?rinar* or genital or vesico* or vaginal or cytovaginal) adj3 fistul*).ab,kw,ti. | 7442 |
| 3 | (((midvaginal or juxtacervical) adj3 obstetric vesicovaginal fistul*) or VVF).ab,kw,ti. | 910 |
| 4 | 1 or 2 or 3 [Genitourinary Fistula] | 8678 |
| 5 | exp surgery/ | 6488388 |
| 6 | (surg* or repair* or reconstruct*).ab,kw,ti. | 4107982 |
| 7 | 5 or 6 [Repair] | 7835542 |
| 8 | 4 and 7 [Genitourinary Fistula and Repair] | 6724 |
| 9 | limit 8 to conference abstracts | 1722 |
| 10 | 8 not 9 [Conference abstracts excluded] | 5002 |
| 11 | (exp animal/ not human/) or animal disease/ or exp experimental animal/ or exp animal experiment/ or exp animal model/ or exp domestic cattle/ or exp rodent/ | 7664251 |
| 12 | ((animal adj3 (model* or experiment*)) or canine or dog or dogs or beagle* or feline or cat or cats or rodent* or rabbit* or mice or mouse or murine* or rat or rats).ab,kw,ti. | 4948279 |
| 13 | 11 or 12 [Animal filter] | 8302970 |
| 14 | 10 not 13 [Animals excluded] | 4929 |
| 15 | exp case study/ | 109694 |
| 16 | (case adj3 (stud* or series)).ab,kw,ti. | 564061 |
| 17 | 15 or 16 [Case reports] | 588151 |
| 18 | 14 not 17 [Case reports excluded] | 4815 |
| 19 | ("23201329" or "22914460" or "27821123" or "33164206" or "33811637" or "31778211" or "29441572" or "27741181" or "26233699" or "20700729").pm. | 10 |
| 20 | limit 18 to yr="2022 -Current" | 326 |

| **Source:** | Search in:Web of Science Core CollectionEditions:All |
| --- | --- |

Date of search: 19-01-2022

| # | Searches | Results |
| --- | --- | --- |
| 1 | TS=(obstetric fistul* or genito?rinar fistul* or genital fistul* or vesicovaginal fistul* or vaginal fistul* or cytovaginal fistul*) | 4.154 |
| 2 | TS = ((midvaginal or juxtacervical NEAR/3 obstetric vesicovaginal fistul*) or VVF) | 425 |
| 3 | #1 OR #2 | 4.269 |
| 4 | TS = (surg* or reconstruct*) | 2.500.403 |
| 5 | #3 AND #4 | 2.408 |
| 6 | TS= (case serie* or case report* or case stud*) | 3.236.511 |
| 7 | #5 NOT #6 | 1.511 |
| 8 | TS = animal* | 1.239.937 |
| 9 | TS = human* | 4.122.819 |
| 10 | #8 NOT #9 | 903.319 |
| 11 | TS = (animal disease OR experimental animal OR animal experiment OR animal model OR domestic cattle OR rodent OR canine OR dog OR dogs OR beagle* OR feline OR cat OR cats OR rodent* OR rabbit* OR mice OR mouse OR murine* OR rat or rats) | 4.323.816 |
| 12 | #10 OR #11 | 4.606.636 |
| 13 | #7 NOT #12 | 1.476 |

Date of update: 20-03-2024

| **#** | **Searches** | **Results** |  |
| --- | --- | --- | --- |
| 1 | TS=(obstetric fistul* or genito?rinar fistul* or genital fistul* or vesicovaginal fistul* or vaginal fistul* or cytovaginal fistul*) | 4621 |  |
| 2 | TS = ((midvaginal or juxtacervical NEAR/3 obstetric vesicovaginal fistul*) or VVF) | 498 |  |
| 3 | #1 OR #2 | 4761 | |
| 4 | TS = (surg* or reconstruct*) | 2913124 |  |
| 5 | #3 AND #4 | 2691 |  |
| 6 | TS= (case serie* or case report* or case stud*) | 3804200 |  |
| 7 | #5 NOT #6 | 1664 |  |
| 8 | TS = animal* | 1390695 |  |
| 9 | TS = human* | 4662268 |  |
| 10 | #8 NOT #9 | 1005928 |  |
| 11 | TS = (animal disease OR experimental animal OR animal experiment OR animal model OR domestic cattle OR rodent OR canine OR dog OR dogs OR beagle* OR feline OR cat OR cats OR rodent* OR rabbit* OR mice OR mouse OR murine* OR rat or rats) | 4684690 |  |
| 12 | 12: #10 OR #11 | 5001249 |  |
| 13 | #7 NOT #12 | 1625 |  |
| 14 | #7 NOT #12 | **148** |  |
