# Supplementary material for: Consensus Statements on the Definition of Surgical Success Following Obstetric Urinary Pelvic Floor Fistula Repair: An IUGA-ICS Proposal
Source: Int Urogynecol J. 2026 Mar 18;37(5):1193–205. doi: 10.1007/s00192-025-06413-6 (PMC13226361; doi:10.1007/s00192-025-06413-6)
Supplement: Supplementary file 2 — Supplementary file2 (DOCX 49.5 KB) [file 192_2025_6413_MOESM2_ESM.docx]

**Identification of studies via databases and registers**

Records identified from:

Medline (n = 3666)

OVID Embase (n = 4788)

Web of Science (n = 1624)

Records removed *before screening*:

Duplicate records removed (n =3137)

**Identification**

Records excluded

Not an original study (n = 895)

Not a human study (n = 14)

No obstetrical vesicovaginal fistula (n = 5183)

No surgical repair (n = 304)

Study size n<10 (n = 72)
 No outcome defined or assessed (n = 2)

Records screened

(n = 6941)

Reports not retrieved

(n = 44)

Reports sought for retrieval

(n = 471)

**Screening**

Reports excluded:

Not an original study (n = 24)

No obstetrical vesicovaginal fistula (n = 43)

No surgical repair (n = 12)

Study size n<10 (n = 32)
 No outcome defined or assessed (n = 174)

Reports assessed for eligibility

(n = 427)

Studies included in review

(n = 142)

**Included**

*From:*  Page MJ, McKenzie JE, Bossuyt PM, Boutron I, Hoffmann TC, Mulrow CD, et al.
The PRISMA 2020 statement: an updated guideline for reporting systematic reviews. BMJ 2021;372:n71.
doi: 10.1136/bmj.n71. For more information, visit: <http://www.prisma-statement.org/>
