# Supplementary material for: Consensus Statements on the Definition of Surgical Success Following Obstetric Urinary Pelvic Floor Fistula Repair: An IUGA-ICS Proposal
Source: Int Urogynecol J. 2026 Mar 18;37(5):1193–205. doi: 10.1007/s00192-025-06413-6 (PMC13226361; doi:10.1007/s00192-025-06413-6)
Supplement: Supplementary file 3 — Supplementary file3 (DOCX 16.8 KB) [file 192_2025_6413_MOESM3_ESM.docx]

**Appendix A: Biosketch and credentials of contributors**

**Dr. Lennart Maljaars** is a medical doctor and researcher from the Amsterdam University Medical Center in the Netherlands. He wrote his PhD thesis under guidance of Prof. J.P.W.R. Roovers at the University of Amsterdam and Prof. D. Bezuidenhout at the University of Cape Town, South-Africa. His research focusses on surgical care and innovation for vesicovaginal fistula repair and includes the development of tissue-engineering techniques for surgical closure of vesicovaginal defects. He has published multiple peer-reviewed papers on (obstetric) vesicovaginal fistula and has initiated this consensus group as a member of International Urogynecology Association (IUGA). Dr. Maljaars is currently a resident in Obstetrics and Gynecology at the University Medical Center Utrecht.

**Prof. Dr. Jacques Corcos** is a Professor of Urology at McGill University and a practicing urologist at the Jewish General Hospital, a McGill-affiliated teaching hospital in Montreal. In 2011, he founded the *"Fondation Mères du Monde en Santé"* (FMMS), a Canadian NGO dedicated to helping eradicate fistulas worldwide. Since its inception, FMMS has organized over 20 fistula camps in various African countries, with Dr. Corcos leading 16 of these missions. Through these initiatives, more than 400 patients have received surgical treatment. Dr. Corcos has also developed several fistula prevention programs and research protocols in the region. He is currently working on the construction of a fistula hospital in Togo. From 2008 to 2011, he served as the General Secretary of the International Continence Society (ICS), where he played an active role in establishing the Fistula Committee within the organization.

**Prof. Dr. Gamal Ghoniem** is currently Professor Emeritus and Vice-Chair at the UC Irvine Health School of Medicine Department of Urology, and Fellowship director, Division of Female Urology and Voiding Dysfunction. He is American Board certified in Urology (ABU) and ABU/certified in the subspeciality of Female Urology, now Urogynecology and pelvic reconstructive surgery-UPRS. He has extensive experience in treating simple and complex vesicovaginal fistula. He is a frequent visiting consultant to Egypt and performs multiple vesicovaginal fistulas repairs. He is expert in female pelvic reconstructive surgery. He has several publications in vesicovaginal fistula: including the International Continence Society (ICS) report on the terminology of female pelvic floor fistulas.

**Prof. Dr. Judith Goh** is a urogynaecologist, practicing in Queensland, Australia, at the Greenslopes Private Hospital and at the Gold Coast University Hospital. Since 1995, Prof. Goh has been a medical volunteer, working mainly in the area of obstetric fistula. She had the privilege of working with Dr. Catherine Hamlin at the Addis Ababa Hospital and was a staff fistula surgeon at this hospital in 1997. Over the past 30 years, Prof. Goh has spent at 8-10 weeks a year in various regions in Asia and Africa, mainly focused on pelvic floor fistula management and has performed/taught over 3000 fistula repairs. Her fistula classification has been utilized over the past 20 years. Prof. Goh was a committee member of the ICS Terminology for Pelvic Floor Fistula (2020) and a member of the Fistula Committee on the International Consultation on Incontinence (2022). In February 2024, she was invited to participate at the FIGO Fistula Expert Surgical Workshop at the Addis Ababa Fistula Hospital. In 2012, she was awarded the Officer of the Order of Australia (AO) for “distinguished service to gynecological medicine, particularly in the field of fistula surgery, and to the promotion of the rights of women and children in developing countries”.

**Dr. Tamsin Greenwell** is a urologist and Associate Professor of Urology practicing in London, United Kingdom at University College London Hospitals (UCLH). She has a completely subspecialized practice in female and male urinary tract reconstruction including fistula surgery and is lead for the 7 urologist section of Functional, Reconstructive and Adolescent Urology at UCLH. She runs the annual 3-day UCLH Female Urology and Urogynaecology live surgery Masterclass during which she has demonstrated vaginal repair of VVF many times. She currently has the largest urinary tract fistula practice in the United Kingdom and has published extensively on this subject. She travels to Lilongwe in Malawi annually with British Association of Urology’s “Urolink” philanthropic section to mentor the local urologists and gynecologists in pelvic floor reconstruction and incontinence surgery. She was the RCS(Eng) Urology Tutor, the BAUS Director of Education, a Member of the Board of the AUA’s GURS section and UCL Postgraduate Tutor in Urology. She is currently the Chair of the United Kingdom Continence Society, a Committee Member for the European Association of Urology Reconstructive Section (ESGURS), a member for the European Association of Urology Video Congress Committee and a member of the ICS committees on The Joint Terminology for Female Lower Urinary Tract, Pelvic Floor Function and Dysfunction and The ICS Global Urodynamics Data Repository Steering Group.

**Dr. David Kupualor** is an obstetrician and gynecologist who has been practicing over the past 8 years. With a great desire to improve the quality of life of patients he joined the subspecialty training in Urogynecology 4 years ago. He is currently a Urogynaecology Fellow with the Korle Bu Teaching Hospital, Accra, Ghana. He is a former Medical Director of the St Joseph Hospital, Koforidua, Eastern Region, Ghana (2017 to 2021) and St John of God Hospital, Asafo, Western North, Ghana (2010 to 2012). He has been actively involved in the management of genitourinary and rectovaginal fistulas over the past 4 years. He is currently part of a fistula outreach team from the Obstetrics and Gynecology unit of Korle Bu Teaching Hospital that performs fistula surgeries on monthly basis at the Mercy Women’s Catholic Hospital, Mankessim in the Central Region of Ghana which is about 2 hours drive from the capital, Accra. With the support of the Fistula Foundation, the local team performs about 15 obstetric and gynecological fistula surgeries in Mankessim, Ghana every month. Aside being a Urogynaecology Fellow, he holds a Master in Business Administration (MBA) from the University of Ghana, Legon and a certificate in Health Administration and Management from the Ghana Institute of Management and Public Administration (HAM-GIMPA). With an early interest in publishing, he was the editor for a health magazine produced by the Kwame Nkrumah University of Science and Technology Medical Students Association (KNUST-MSA) in 2005 as well as the School of Medical sciences (SMS), KNUST Class of 2007 yearbook.

**Dr. Rachel Pope** is Associate Professor of Obstetrics and Gynecology and Urology at the University Hospitals Cleveland Medical Center Urology Institute. She has been studying women with obstetric fistulas from a public health perspective for the last 20 years. She is an expert fistula surgeon who focuses her work on reconstructing the vagina for long term sexual function as well as repairing the fistula. She is the chief of Female Sexual Health at her institute, a leader in the Roe Green Global Health program, the FIGO Chair of the Obstetric Fistula committee, and teaches Women’s Public Health at Case Western Reserve University.

**Dr. Suneetha Rachaneni** is a consultant gynecologist and Accredited Subspecialist in Urogynaecology at Shrewsbury and Telford Hospitals NHS Trust, Shropshire, UK. After graduating from the University of Health Sciences in India, she completed specialist training in the Southwest Peninsula and the West Midlands Regions. Dr. Rachaneni is the clinical lead for Urogynaecology at Shrewsbury and Telford Hospitals Trust. Dr. Rachaneni takes a keen interest in research and has published several peer reviewed papers. She was a R&D member in IUGA for 6 years and currently serves in the R&D committee of British Society of Urogynaecology

**Prof. Dr. Mohan Chandra Regmi** is Professor of Obstetrics and Gynecology in BP Koirala Institute of Health Sciences in Eastern Nepal. This is one of the biggest university hospitals in Nepal. He also looks after the Urogynecology Fellowship program supported by Foundation of International Urogynecology Assistance ([www.fiuga.org](http://www.fiuga.org)). He is also National Trainer for Obstetric Fistula. The institute has been termed as National Centre for Obstetric Fistula. The on-the-job training module of Obstetric Fistula has been integrated into Urogynecology Curriculum. The graduating fellows are able to carry out fistula repairs adding sustainability of fistula repair services a practice which has been followed around the world. Dr. Regmi is also the treasurer of International Society of Obstetric Fistula Surgeons (ISOFS). He is responsible for leading the society’s fight against fistula in South Asian region. He is supporting the different fistula centers in South Asian region with live surgeries, workshops and dissemination of experience. He is committed to improve maternal health in South Asia.
